# HIGH-DEDUCTIBLE HEALTH PLANS AND COST OF HEARING AIDS AMONG ADULTS WITH HEARING LOSS: IMPLICATIONS FOR DISPARITIES

**DOI:** 10.1093/geroni/igz038.929

**Published:** 2019-11-08

**Authors:** Elham Mahmoudi, Neil Kamdar

**Affiliations:** 1 University of Michigan, Department of Family Medicine, Ann Arbor, Michigan, United States; 2 University of Michigan, Ann Arbor, Michigan, United States

## Abstract

High-deductible health plans (HDHPs) have shown potential to curb rising healthcare costs. We examined use and cost of hearing aids (HAs), comparing HDHPs with non-HDHPs. Using the 2009-2016 Truven Marketscan claims, we identified adults aged 50-64 who were diagnosed with hearing loss (HL) and whether they used HAs or not (n=1,247,113). We applied multivariable generalized linear models, adjusting for age, gender, hierarchical condition categories (HCCs). To control for potential selection bias, we applied an inverse propensity score weighting. Our outcomes of interest included: (1) utilization and (2) total and out-of-pocket costs of HAs (inflation adjusted to 2016 dollars), comparing HDHPs with non-HDHPs. Number of enrollees in HDHPs increased by 343%, from 1,717 in 2009 to 7,615, in 2016. The percentage of patients who used HA increased from 9.5% (95% CI:0.09-0.10) to 15% (95% CI:0.14-0.15) within non-HDHPs and from 5% (95% CI:0.04-0.06) to 16% (95% CI:0.15-0.17) within HDHPs. The average adjusted cost sharing and total cost of HAs increased by 74% ($85 to $148) and 52% ($589 to $894), respectively, among non-HDHPs; they increased by 80% ($173 to $312) and 91% ($589 to $1,126), respectively, among HDHPs. Average out-of-pocket costs for HAs in HDHPs were twice as much as in non-HDHPs (p <0.0001). Total and out-of-pocket costs for hearing aids were substantially higher among HDHPs compared with non-HDHPs. Many employers have started offering only HDHPs, leaving their employees with no other health insurance option. Higher cost sharing may worsen the existing socioeconomic disparities in access to HAs.

